# Proton pump inhibitors modulate esophageal epithelial barrier function and crosstalk with eosinophils

**DOI:** 10.1111/pai.70315

**Published:** 2026-02-27

**Authors:** Ravi Gautam, Megha Lal, Margaret C. Carroll, Zoe Mrozek, Tina Trachsel, Jarad Beers, Melanie A. Ruffner

**Affiliations:** ^1^ Division of Allergy and Immunology Children's Hospital of Philadelphia Philadelphia Pennsylvania USA; ^2^ Division of Allergy University Children's Hospital Zurich Zurich Switzerland; ^3^ Department of Pediatrics Perelman School of Medicine at University of Pennsylvania Philadelphia Pennsylvania USA

**Keywords:** chemokine, eosinophil, eosinophilic esophagitis, epithelial barrier, proton pump inhibitor

## Abstract

**Background:**

Eosinophilic esophagitis (EoE) is a chronic allergic disorder driven by type 2 inflammation, characterized by eosinophilic infiltration and esophageal epithelial abnormalities, including barrier dysfunction, basal cell hyperplasia, epithelial thickening, and loss of differentiation. Although proton pump inhibitor (PPI) therapy is frequently employed in the management of EoE and is known to reduce esophageal eosinophilia, improve barrier function, and exert anti‐inflammatory effects, the precise mechanism by which PPIs modulate type 2 inflammation and epithelial integrity remains incompletely understood.

**Methods:**

Air‐liquid interface culture of esophageal epithelial cells was used to investigate the impact of the PPI omeprazole on barrier integrity in IL‐13‐treated cultures. Epithelial chemokine secretion was assessed following stimulation with IL‐13 and omeprazole, and eosinophil migration from healthy human donors was evaluated using 3 μm pore‐sized transwells. A co‐culture system of epithelial cells and eosinophils was used to examine chemokine secretion, eosinophil adhesion, and activation marker expression.

**Results:**

Omeprazole treatment of IL‐13‐treated air‐liquid interface (ALI) cultures restored barrier integrity compared with IL‐13‐treated ALI cultures and resulted in 186 differentially expressed genes. Omeprazole treatment reduced STAT6 phosphorylation, downregulated calpain 14 expression, and upregulated desmoglein‐1 in IL‐13‐treated ALI samples. IL‐13 treatment induced upregulation of Eotaxin‐3, CXCL10, and periostin, which were downregulated by omeprazole. Eosinophils co‐cultured with human esophageal epithelial cells in the presence of omeprazole had diminished CD11b, CD18, and CD69 expression compared to those cultured with IL‐13 alone, and less eotaxin‐3, CXCL10, CCL2, and CCL4 were recovered from the co‐culture media.

**Conclusion:**

Omeprazole diminished the effects of IL‐13 in both the epithelial air‐liquid interface model and eosinophil‐epithelial co‐cultures, alleviating barrier dysfunction, chemokine expression, and the upregulation of eosinophil adhesion markers.

**Key message**

Eosinophilic esophagitis (EoE) is a chronic, immune‐mediated disease characterized by esophageal inflammation and dysfunction. Proton pump inhibitors (PPIs) like omeprazole are widely used in EoE treatment, yet their mechanisms of action remain incompletely understood. This study investigated omeprazole's effects on IL‐13‐treated esophageal epithelial cells and eosinophil interactions. Omeprazole restored epithelial barrier integrity, reduced STAT6 phosphorylation, and modulated key gene expression, including downregulation of CAPN14 (calpain‐14) and upregulation of Dsg1 (desmoglein‐1). It also diminished IL‐13‐induced epithelial chemokine secretion and reduced eosinophil adhesion and activation markers. These findings suggest that omeprazole mitigates IL‐13‐driven epithelial dysfunction and eosinophilic inflammation, highlighting its potential therapeutic role in EoE.

## 1 INTRODUCTION

Eosinophilic esophagitis (EoE) is a chronic immune‐mediated disorder triggered by specific food antigens, characterized by eosinophil‐rich multicellular inflammation and epithelial changes.^1^ Treatment options for EoE include dietary therapy, pharmacological therapy, such as proton pump inhibitors (PPIs), corticosteroids, and biologic therapy.^2^

Proton pump inhibitors (PPIs), initially developed as acid‐suppressive agents for reflux and peptic ulcer disease, have become a mainstay of EoE therapy. Although historically used to distinguish EoE from gastroesophageal reflux disease (GERD), PPI trials are no longer used after studies showed that PPIs resolve the type 2 inflammatory signature in EoE in patients who achieve histologic remission on PPI therapy.^3^ Instead, attention has shifted to understanding their therapeutic mechanisms. PPIs inhibit ATP12A, reduce phosphorylated STAT6 binding and eotaxin‐3 expression, and upregulate the aryl hydrocarbon receptor pathway, collectively dampening type 2 inflammation.^4^, ^5^, ^6^, ^7^ While these pathways explain part of their benefit, important questions regarding the mechanisms of PPI activity remain.

Transcriptomic profiling has begun to shed light on this issue. Although IL‐13 can induce transcriptional programs in esophageal epithelial cells that mimic changes seen in EoE, Rochman et al. demonstrated that PPI treatment in vitro modulates these signatures.^4^ A key effect of IL‐13 is the robust induction of CCL26 (eotaxin‐3), a potent eosinophil chemoattractant, via a STAT6‐dependent transcriptional mechanism.^8^ IL‐13 also induces calpain‐14 (CAPN14), an esophageal‐specific protease, and suppresses desmoglein‐1 (Dsg1), a critical adhesion molecule, which can disrupt epithelial integrity.^9^, ^10^ However, whether these transcriptional changes translate into functional protection of the epithelial barrier and altered immune cell interactions remains unclear, as PPI treatment has been associated with improved barrier function in tissue from EoE patients but unchanged barrier function in vitro.^4^, ^11^ Therefore, understanding whether these transcriptional changes lead to altered epithelial barrier function and mucosal immune signaling remains crucial for fully grasping PPI efficacy in EoE.

This study examines the effects of omeprazole, a PPI commonly used for EoE therapy, on the dynamics of esophageal epithelial barrier function and the crosstalk between the epithelium and eosinophils. We hypothesized that administering omeprazole along with IL‐13 would prevent or reduce epithelial barrier dysfunction and inflammation. To evaluate this, we used the IL‐13‐treated air‐liquid interface (ALI) culture model to examine how omeprazole impacts epithelial barrier integrity, mimicking PPI use in EoE treatment. Additionally, we employed an epithelial‐eosinophil co‐culture system to explore how omeprazole influences epithelial chemokine secretion as well as eosinophil activation and migration.

## 2 METHODS

### 2.1 Esophageal epithelial cell culture

Immortalized human esophageal epithelial cells (EPC2‐hTERT, EPC2) were cultured in keratinocyte serum‐free media (KSFM, Thermo Fisher Scientific, USA) containing 0.09 mM Ca^2+^, 1 ng/mL recombinant human epidermal growth factor, 50 μg/mL bovine pituitary extract, and 100 U/mL penicillin and streptomycin at 37°C in a humidified 5% CO_2_ incubator. Cells were grown in KSFM with 1.8 mM Ca^2+^ for 48 h to promote maturation and differentiation, then stimulated with 100 ng/mL recombinant human IL‐13 (SRP3274, Sigma–Aldrich) or 50 μM acid‐activated omeprazole (O104, Sigma–Aldrich).

### 2.2 Air‐liquid interface (ALI) culture

EPC2 cells were cultured on 0.4 μm transwells (Corning Life Science) in KSFM for 3 days, then switched to 1.8 mM Ca^2+^ KSFM for 5 days.^12^ On day 7, the upper chamber media was removed to promote terminal differentiation. Between days 10–14, IL‐13 (100 ng/mL), 50 μM acid‐activated omeprazole, or both were added to the basolateral chamber. On day 14, each 24 mm insert (4.67 cm^2^ growth area) was cut into two halves, one used to assess Dsg1 and pSTAT6 protein, and the other half for measuring CAPN14 mRNA expression. For experiments evaluating effects of STAT6 inhibitor, AS1517499 (500 nM, Sigma Aldrich) was pretreated 2 h before IL‐13.

### 2.3 Bulk RNA sequencing and data analysis

ALI cultures were submerged in TriReagent for RNA isolation (Qiagen). RNA sequencing libraries were prepared (Illumina TruSeq RiboZero), followed by single‐end sequencing (Illumina NovaSeq 6000). Raw sequencing data are available in the NCBI GEO series database (accession GSE273039).^13^ Please see the Appendix S1 for RNA‐seq data analysis workflow.

### 2.4 Transepithelial electric resistance (TEER)

Resistance across the ALI was measured using a Millicell ERS‐2 Voltohmeter (Merck Millipore) on days 10, 12, and 14. TEER for each well was calculated by subtracting blank transwell resistance and multiplying by surface area (0.33 cm^2^ for 24‐well inserts). Samples with TEER ≤200 Ω*cm^2^ on day 10 were excluded from stimulation experiments.

### 2.5 Paracellular flux assay

On day 14, 70 kDa FITC‐dextran (3 mg/mL, Sigma‐Aldrich) was added to the upper chambers and incubated at 37°C for 4 h. Basolateral media were collected and fluorescence measured at Ex/Em = 485/520 nm using a Thermo Scientific Varioskan™ LUX microplate reader. Sample concentrations were determined via a standard titration curve (30–0.23 μg/mL).

### 2.6 ALI histology and immunofluorescence

On day 14, ALI cultures were formalin‐fixed, paraffin‐embedded, serially sectioned, and stained with hematoxylin and eosin.

For immunofluorescence, slides were deparaffinized, permeabilized, blocked with 5% BSA, then incubated overnight with mouse anti‐Dsg1 (1:50, Santa Cruz Biotechnology) at 4°C. Sections were rinsed and incubated for 1 h with Donkey anti‐Mouse IgG AF488 (Thermo Scientific) and DAPI (Sigma Aldrich).

Immunohistochemistry was performed using the Leica BOND Rxm automated slide stainer with heat‐induced epitope retrieval solution 2 (HIER2). Sections were incubated with a rabbit anti‐TP63 antibody (1:500, Abcam), and detection was performed using the Bond Polymer Refine Detection Kit (Leica Biosystems). Images were acquired with the EVOS M700 imaging system (Thermo Fisher Scientific). To quantify TP63^+^ nuclei, three 40X regions of interest (ROIs) per image from four ALI cultures were analyzed. Nuclei counting was performed using the Color Deconvolution 2 and Cell Counter ImageJ plugins.

### 2.7 Quantitative real‐time PCR (qRT‐PCR)

RNA was isolated, and cDNA was synthesized from 1 μg of RNA using iScript™ Reverse Transcription Supermix for RT‐qPCR (Bio‐Rad). qRT‐PCR was performed using 25 ng cDNA with SsoAdvanced™ Universal SYBR® Green Supermix (Bio‐Rad) and primers for CAPN14 (Bio‐Rad assay ID: qHsaCID0017001) and GAPDH (qHsaCED0038674).

### 2.8 Western Blot

Cell lysates (15 μg protein) were resolved on 12% polyacrylamide gels, transferred to 0.2 μm PVDF, blocked, and incubated overnight at 4°C with primary antibodies (Mouse anti‐STAT6, 1:1000, sc‐374,021, Santa Cruz; Rabbit anti‐pSTAT6, 1:1000, SAB4300038, Sigma‐Aldrich; Mouse anti‐Dsg1, 1:1000, sc‐137,164, Santa Cruz; Mouse anti‐ICAM‐1, 1:500, MA5407, Invitrogen). Blots were washed, incubated for 1 h with secondary antibodies (StarBright Blue 520 goat anti‐mouse and StarBright Blue 700 goat anti‐rabbit, 1:2500, Bio‐Rad or anti‐rabbit IgG HRP‐linked HRP, 1:2500, Cell Signaling Technology), and probed for tubulin or actin (hFAB Rhodamine, 1:2500, Bio‐Rad). Proteins were visualized on a ChemiDoc system (Bio‐Rad) using either fluorescent detection or Clarity Max™ ECL substrate (Bio‐Rad) and analyzed with Image Lab software.

### 2.9 Eosinophil isolation and culture with EPC2 cells

Whole blood was collected from healthy human donors with informed consent, approved by The Children's Hospital of Philadelphia Institutional Review Board. Eosinophils were isolated via density gradient separation, red cell lysis with ammonium chloride, and immunomagnetic negative selection for CD45 + CD66b + CD16‐ eosinophils (EasySep™, STEMCELL). Purity (>99%) was confirmed by flow cytometry (Figure S1).

For eosinophil monocultures, media was supplemented with IL‐5 (10 ng/mL, Peprotech) to ensure viability. For co‐culture experiments, eosinophils (3 × 10^5^) were added to confluent EPC2 cells in a 24‐well plate with a 1:1 mix of high‐Ca^2+^ KSFM and RPMI (10% FBS). Cultures were treated for 24 h with IL‐13, omeprazole, or both, then cells and culture supernatants were harvested for analysis.

### 2.10 Multiplex and ELISA

Cell culture supernatants from the co‐culture were analyzed using a multiplex cytokine discovery assay (Eve Technologies). Eotaxin‐3, CXCL10, periostin, CCL2, and CCL4 concentrations were subsequently quantified by ELISA (R&D Systems) per the manufacturer's instructions. Optical density was read at 450/570 nm using a Thermofisher multimode microplate reader.

### 2.11 Eosinophil migration assay

Freshly isolated eosinophils were added to the upper chambers of 3 μm transwells (Corning). Conditioned media from EPC2 cultures or fresh culture media supplemented with IL‐5 (5 ng/mL) were placed in the lower chambers. After 5 h, migrating eosinophils were counted from the lower chamber.

### 2.12 Flow cytometry

Eosinophils were stained for viability (Zombie NIR™, Biolegend) in PBS for 15 min, washed, then incubated with Fc Block (BD Bioscience) for 15 min. Cells were stained with BV605 anti‐CD45, FITC anti‐CD18, BV785 anti‐CD11b, and BV421 anti‐CD69 (BioLegend). Data were acquired using an LSR‐Fortessa and analyzed with FlowJo (BD Biosciences).

### 2.13 Statistical analysis

Statistical analyses were performed using *t*‐tests for two‐group comparisons and one‐way ANOVA with Tukey's post hoc test for multiple comparisons. *p‐*values <.05 were considered significant. Analyses were conducted in GraphPad Prism (version 10.2.3).

## 3 RESULTS

### 3.1 Omeprazole modulated IL‐13‐induced transcriptional changes in epithelium

**FIGURE 1:**
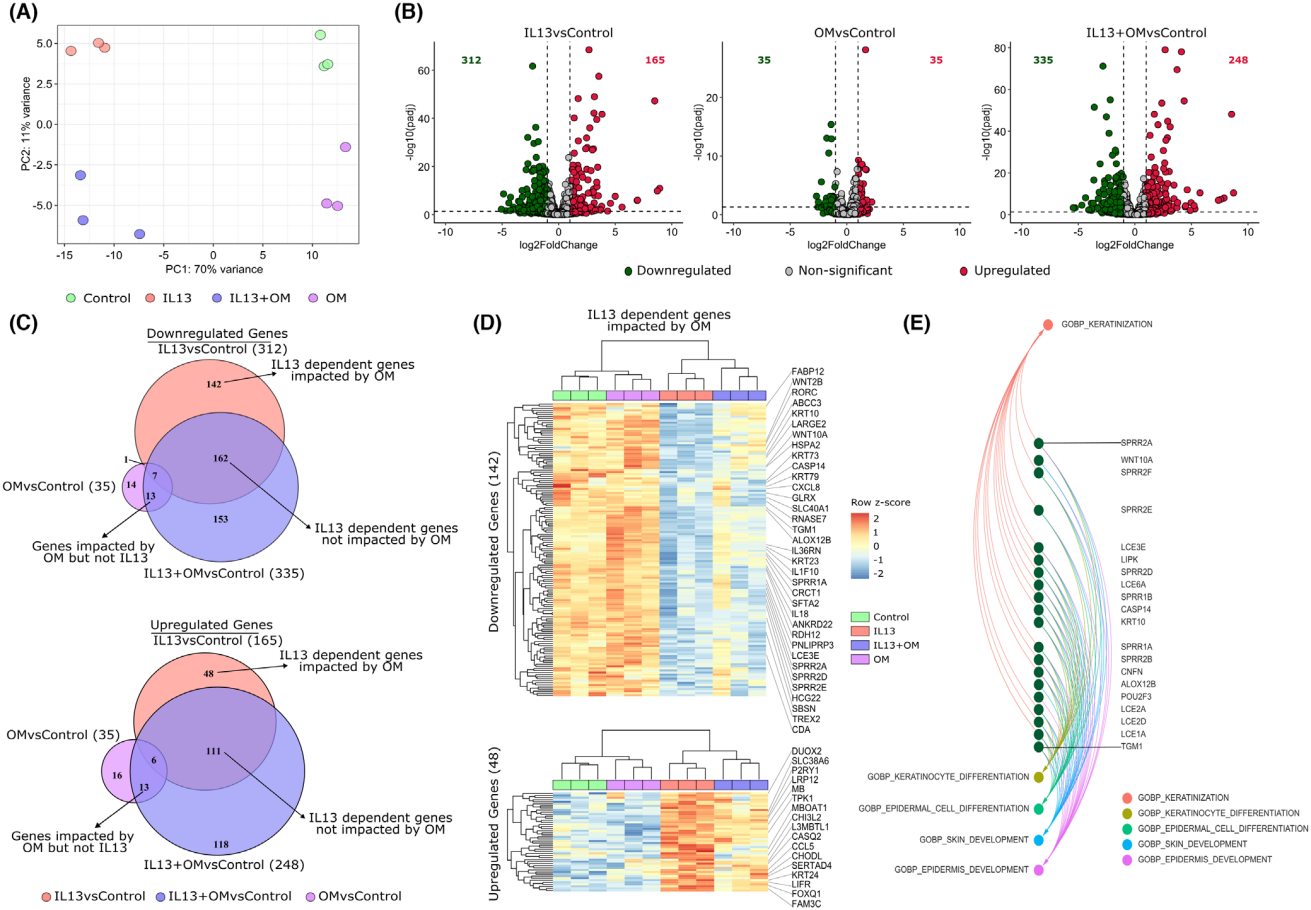
Omeprazole treatment altered differential gene expression in IL‐13‐treated esophageal epithelial cells at the air‐liquid interface (ALI). (A) Principal Component Analysis (PCA) of the top 500 most variable genes revealed clustering of samples by treatment condition. (B) Volcano plot of differentially expressed genes (DEGs), displaying log_2_ (Fold Change) versus statistical significance (−log_10_ adjusted *p*‐value). Significant genes are identified at a false discovery rate (FDR) < 0.05 and log_2_FC ≥ |1|. (C) Venn diagram comparing the downregulated (top) and upregulated (bottom) genes in three groups: IL13 versus Control, OM (omeprazole) vs. Control and IL‐13 + OM vs. Control. (D) Heatmap of genes downregulated or upregulated by IL‐13 and impacted by OM (E) Interaction network plot displaying biological pathways associated with the IL‐13‐dependent genes that are affected by omeprazole.

We used RNA‐seq to examine the impact of omeprazole treatment in the IL‐13‐treated ALI model of EoE.^14^ Principal component analysis (PCA) of all expressed genes showed that PC1 accounted for 40% of the total variance and separated IL‐13–treated samples from non‐treated samples (Figure S2). Omeprazole‐treated samples did not show clear separation from non‐treated samples when all genes were included in the analysis. When PCA was performed using the 500 most variable genes, PC1 (70% variance) distinguished IL‐13‐treated from non‐treated samples, while PC2 (11% variance) differentiated omeprazole‐treated from nontreated samples (Figure 1A).

Differential expression analysis (FDR <0.05, log_2_FC ≥ |1|) identified 312 downregulated and 165 upregulated genes in IL‐13‐treated samples versus controls (Figure 1B, Table S1). Consistent with prior studies, IL‐13 affected STAT6‐regulated genes linked to EoE (CCL26, TNFAIP6, NTRK1, SERPINB4, CAPN14).^4^ In contrast, omeprazole‐treated samples showed fewer DEGs (35 downregulated, 35 upregulated), suggesting a milder transcriptional impact compared to IL‐13. Compared with control alone, we found 335 downregulated and 248 upregulated genes in combined IL‐13 and omeprazole treatment (Figure 1B). Comparison of the DEGs identified with Rochman et al., who also studied omeprazole and esomeprazole effects on esophageal epithelium, showed limited overlap between datasets, likely reflecting differences in experimental design (Figure S3).

To analyze the effect of omeprazole on IL‐13–mediated transcription, we examined overlapping DEGs across three conditions: IL‐13 vs. Control, Omeprazole vs. Control, and IL‐13 + Omeprazole vs. Control (Figure 1C). This analysis stratified IL‐13–regulated genes into two categories: (i) IL‐13–dependent genes affected by omeprazole (142 downregulated, 48 upregulated), and (ii) IL‐13–dependent genes not impacted by omeprazole (162 downregulated, 111 upregulated) (Figure 1D). In total, 39% of the IL‐13‐responsive genes were affected by omeprazole. We conducted GO term enrichment analysis to identify IL‐13‐regulated pathways affected and unaffected by omeprazole treatment. Genes downregulated by IL‐13 and affected by omeprazole were enriched in pathways related to keratinocyte differentiation and skin development pathways (e.g., WNT10A, TGM1, ALOX12B, KRT10, CASP14, CNFN) (Figure 1E). IL‐13‐downregulated genes with no significant changes elsewhere also showed enrichment in skin development and epidermis development (IVL, FLG, FLG2, CCN2, CALM5, TGM5) (Figure S4).

### 3.2 Omeprazole improved epithelial barrier function in air‐liquid interface (ALI) culture

**FIGURE 2:**
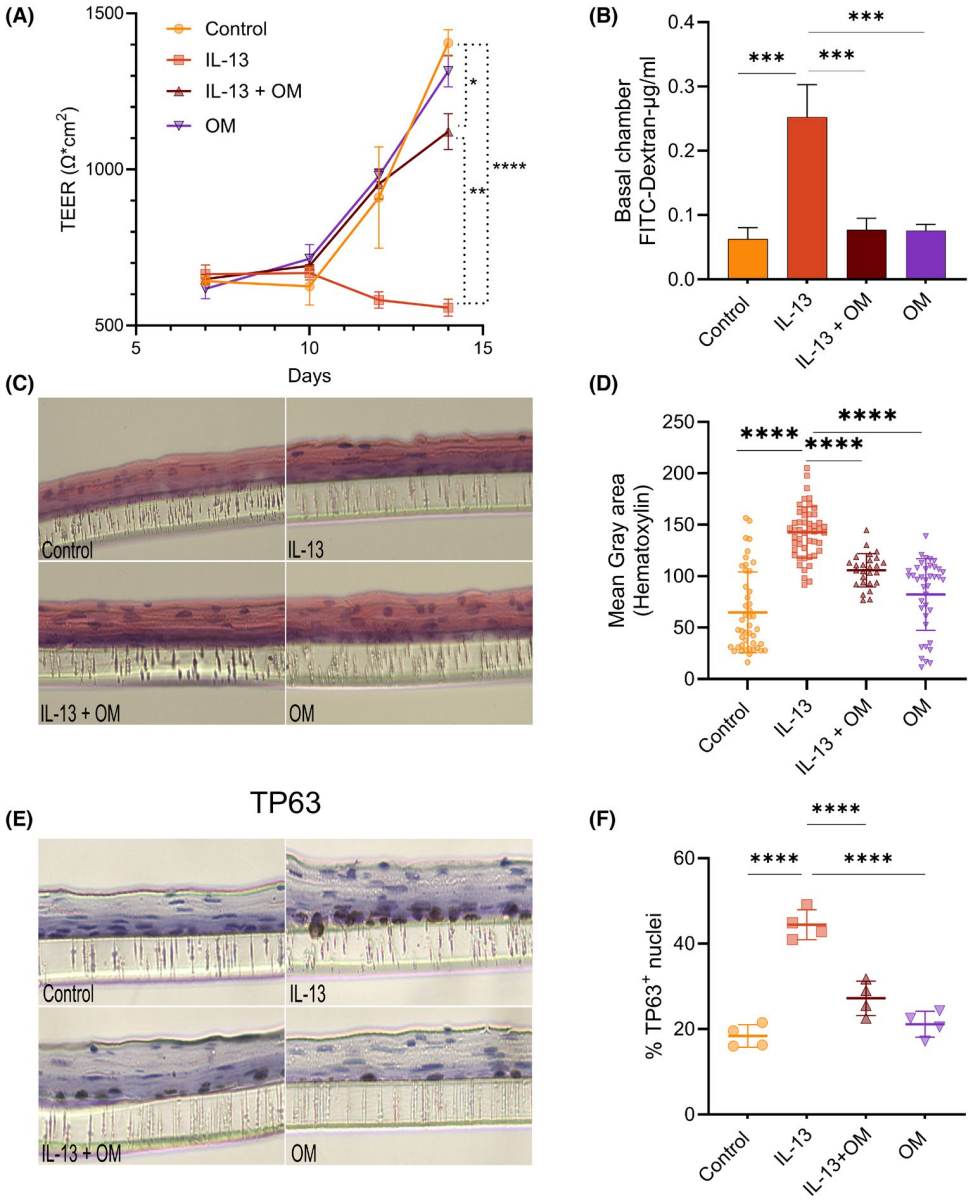
Omeprazole enhanced esophageal epithelial barrier integrity and modulated STAT6 pathway. (A) Transepithelial electrical resistance (TEER) measurements (Ω·cm^2^) in EPC2‐hTERT ALI cultures. Data are presented as mean ± SD (*n* = 3 replicates, representing three independent experiments). (B) Paracellular permeability assay, measuring the translocation of 70 kDa FITC‐Dextran across the ALI membrane into the basolateral chamber. Data are shown as mean ± SD (*n* = 3 replicates, representing two independent experiments). (C) Hematoxylin and eosin (H&E) staining of EPC2‐hTERT ALI cultures illustrating epithelial morphology. (D) Quantification of hematoxylin staining in H&E‐stained ALI cultures (mean ± SD). (E) TP63 immunohistochemistry in ALI cultures. (F) Percentage of TP63^+^ nuclei quantified from TP63 IHC in ALI cultures. Statistical significance is indicated as *p < .05, **p < .01, ***p < .001, ****p < .0001.

As previously described, IL‐13‐treated ALI showed a significant TEER decrease on days 12 and 14 (Figure 2A) and a ~4‐fold increase in FITC‐dextran paracellular flux (Figure 2B), indicating barrier dysfunction. Histological analysis revealed a reduced differentiated layer and basal hyperplasia, with increased basal hematoxylin staining (Figure 2C,D), further confirmed by increased TP63^+^ nuclei (Figure 2E,F). Omeprazole treatment reversed these effects, and we observed improved TEER (though still below control levels) and reduced FITC‐dextran flux, suggesting enhanced barrier integrity (Figure 2A,B). Histologically, omeprazole‐treated ALI exhibited basal and differentiated morphology similar to that of the control (Figure 2C,D). In EoE, basal cell hyperplasia is characterized by an expansion of TP63+ cells.^15^ We found that IL‐13 increases the proportion of TP63+ nuclei in ALI, and omeprazole reversed this effect (Figure 2E,F). These findings suggest that omeprazole mitigates the detrimental effects of IL‐13 on epithelial proliferation and differentiation observed in vitro.

### 3.3 Omeprazole improved barrier integrity by regulating the STAT6 pathway

**FIGURE 3:**
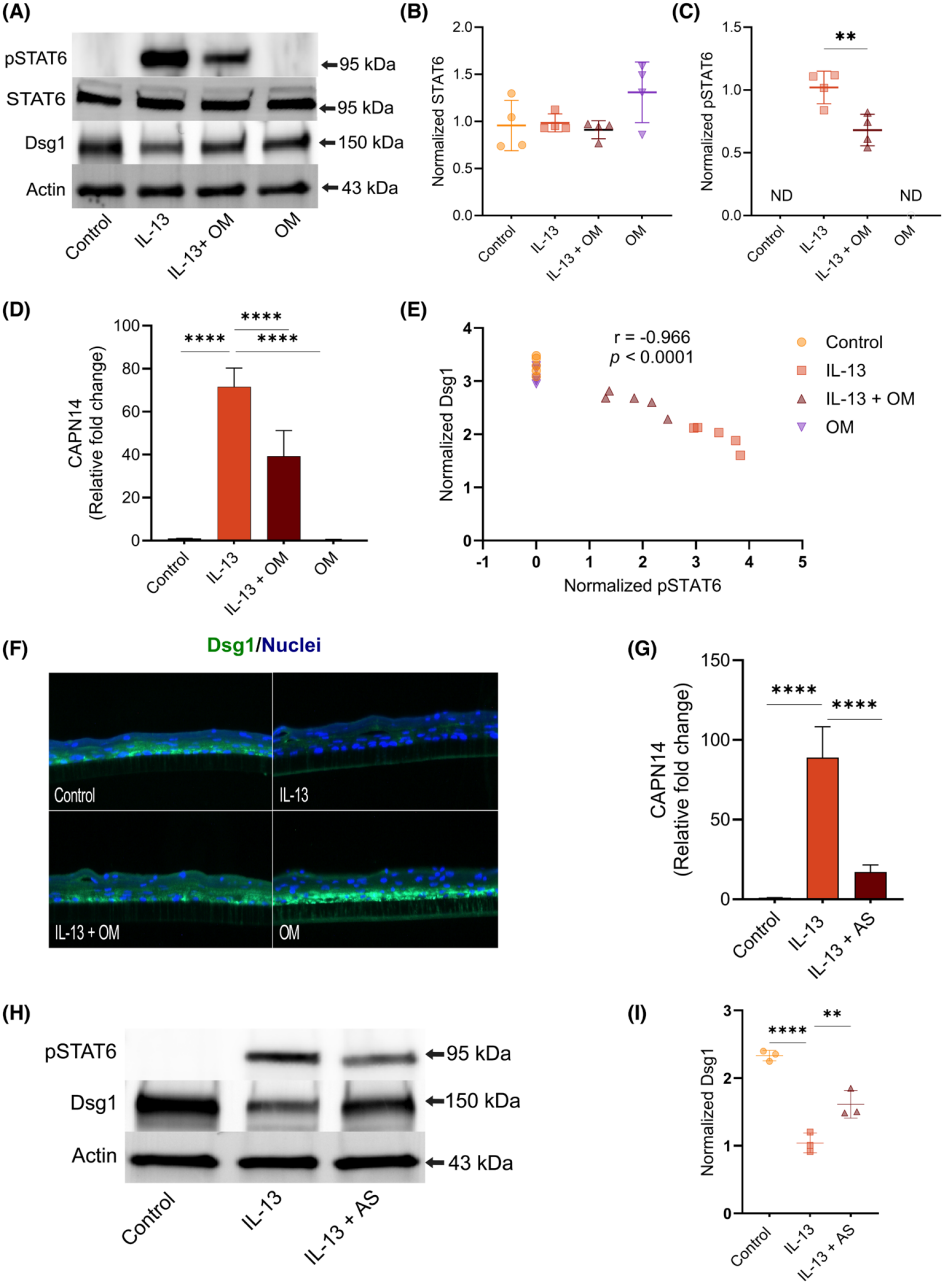
Omeprazole mitigated IL‐13‐induced epithelial remodeling by modulating STAT6 signaling. (A) Representative western blot image showing phosphorylated STAT6 (pSTAT6), total STAT6, and Dsg1. (B) Quantification of STAT6 optical density, normalized to housekeeping protein. (C) Quantification of pSTAT6 optical density. (D) Relative mRNA expression of CAPN14 in ALI cultures, normalized to GAPDH and expressed as fold change relative to control. Data are shown as mean ± SD (*n* = 5). (E) Quantification of normalized pSTAT6 and Dsg1 protein levels from Western blots. Dsg1 expression inversely correlated with pSTAT6 (*n* = 5). (F) 40x image of ALI assessed by Immunofluorescence for desmoglein 1 (Dsg1, green), with nuclei counterstained with DAPI (blue). (G) Relative CAPN14 mRNA expression in IL‐13 ± STAT6 inhibitor (AS1517499, 500 nM) treated cultures (*n* = 3). (H) Representative western blot image showing pSTAT6 and Dsg1 in ALI treated with IL‐13 ± AS1517499 (*n* = 3). (I) Quantification Dsg1 and pSTAT6 protein levels normalized to Actin levels (*n* = 3 replicates from independent experiments). Statistical significance is indicated as **p < .01, ****p < .0001.

IL‐13 treatment leads to a marked upregulation of STAT6 phosphorylation (Figure 3A–C) within the esophageal epithelium. Samples treated with both omeprazole and IL‐13 exhibited a decrease in pSTAT6 expression compared to IL‐13 treated samples, as has previously been described (Figure 3A–C).^5^, ^16^

We hypothesized that omeprazole may impact calpain 14 expression via effects on STAT6 signaling because pSTAT6 has previously been shown to upregulate *CAPN14* expression.^17^ Consistent with prior studies, IL‐13 stimulation robustly induced CAPN14 expression, and we found that omeprazole co‐treatment partially reduced this induction (Figure 3D).

The induction of esophageal epithelial calpain‐14 expression has been tied to loss of desmoglein‐1 and decreased barrier function.^9^, ^18^ We next asked whether these changes were accompanied by changes in desmoglein‐1 (Dsg‐1) expression. Both Western blot analysis and immunofluorescence demonstrated that IL‐13 markedly reduced Dsg‐1 expression, whereas omeprazole co‐treatment significantly restored Dsg1 levels (Figure 3A,F; Figure S5). Importantly, omeprazole alone had no effect on Dsg‐1 expression compared with controls.

Because IL‐13 signals predominantly through the STAT6 pathway, we examined whether omeprazole's effects on desmoglein‐1 expression were associated with changes in STAT6 activation. Quantitative analysis revealed a clear inverse correlation between pSTAT6 and Dsg‐1 expression (Figure 3E), suggesting that STAT6 activation directly contributes to Dsg‐1 downregulation. To test this hypothesis, we blocked STAT6 signaling using the selective inhibitor AS1517499. Inhibition of STAT6 not only prevented the IL‐13–induced upregulation of CAPN14 but also preserved Dsg1 expression, closely mirroring the effects of omeprazole (Figure 3G–I). This indicates that one potential epithelial protective mechanism of omeprazole is to dampen IL‐13–driven STAT6 activation, thereby suppressing CAPN14 induction and maintaining Dsg‐1 expression.

### 3.4 Omeprazole downregulated chemokines responsible for eosinophil migration and survival

Prior work shows that omeprazole impacts pSTAT6 binding at the CCL26 promoter, downregulating eotaxin‐3 in esophageal epithelial cells.^5^ We examined omeprazole's effect on epithelial soluble mediator secretion relevant to eosinophil chemotaxis. Using ELISA, we measured secreted levels of eotaxin‐3, interferon‐gamma inducible protein (IP‐10/CXCL10), and periostin, all of which are associated with eosinophil migration and survival in the epithelium.^19^, ^20^, ^21^, ^22^, ^23^

**FIGURE 4:**
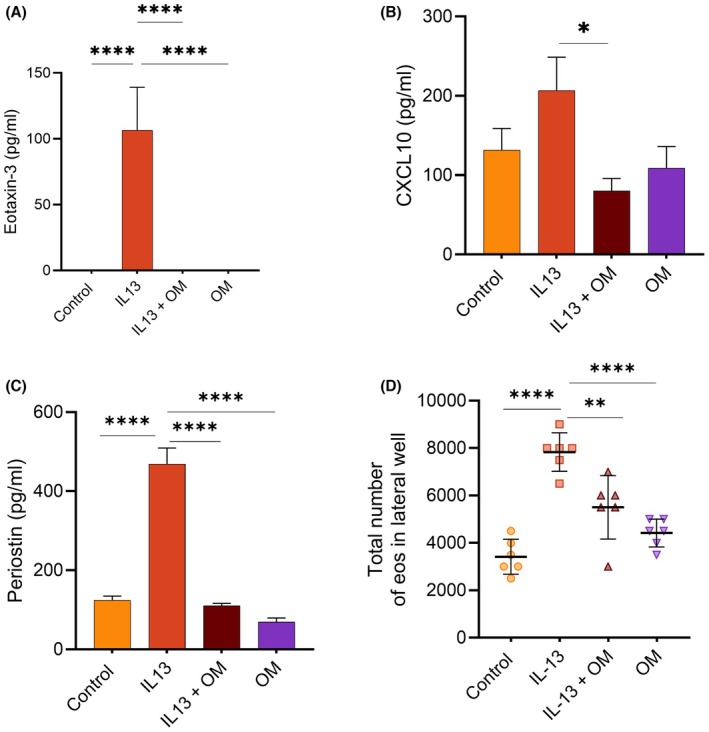
Omeprazole decreased the secretion of inflammatory mediators from esophageal epithelial cells. Levels of (A) eotaxin‐3, (B) CXCL10 (interferon gamma‐induced protein 10, IP10), and (C) periostin were quantified in epithelial culture media using ELISA. (D) Eosinophil (eos) chemotaxis assay measuring migration through transwells in response to conditioned media. Data are presented as mean ± SD (*n* = 6). Statistical significance is indicated as follows: *p < .5, **p < .01, ****<.0001.

IL‐13 stimulation upregulated eotaxin‐3 expression (106.5 ± 32.62 pg/mL) compared to the undetected control level. Eotaxin‐3 was absent in cultures treated with omeprazole alone or with IL‐13 and omeprazole (Figure 4A). Similarly, IL‐13 increased CXCL10 expression ~1.5‐fold compared to the control, but co‐stimulation with IL‐13 and omeprazole significantly reduced CXCL10 expression (Figure 4B). Periostin was significantly higher in IL‐13‐treated cultures (468.9 ± 98.84 pg/mL) versus the control (124.2 ± 25.68 pg/mL). However, periostin levels were significantly lower in IL‐13 and omeprazole co‐treated (110.3 ± 14.54 pg/mL) and omeprazole‐treated (69.47 ± 23.68 pg/mL) cultures (Figure 4C).

To evaluate the overall impact of different chemokine profiles, we performed an eosinophil migration assay using a transwell system. After 5 h, negligible eosinophil migration was observed in media containing IL‐5, IL‐13, omeprazole, or IL‐13 with omeprazole (data not shown). Next, we examined migration toward conditioned media from EPC2 cultures stimulated under various conditions. Maximum eosinophil migration was observed in response to conditioned media from IL‐13‐treated cells, compared to untreated or omeprazole‐treated epithelial cells. Migration in the omeprazole + IL‐13 co‐treatment condition was not increased relative to the control (Figure 4D), supporting our hypothesis that omeprazole reduces chemokine secretion from epithelial cells.

### 3.5 Omeprazole downregulated eosinophil activation markers and ICAM‐1 expression in epithelial cells

**FIGURE 5:**
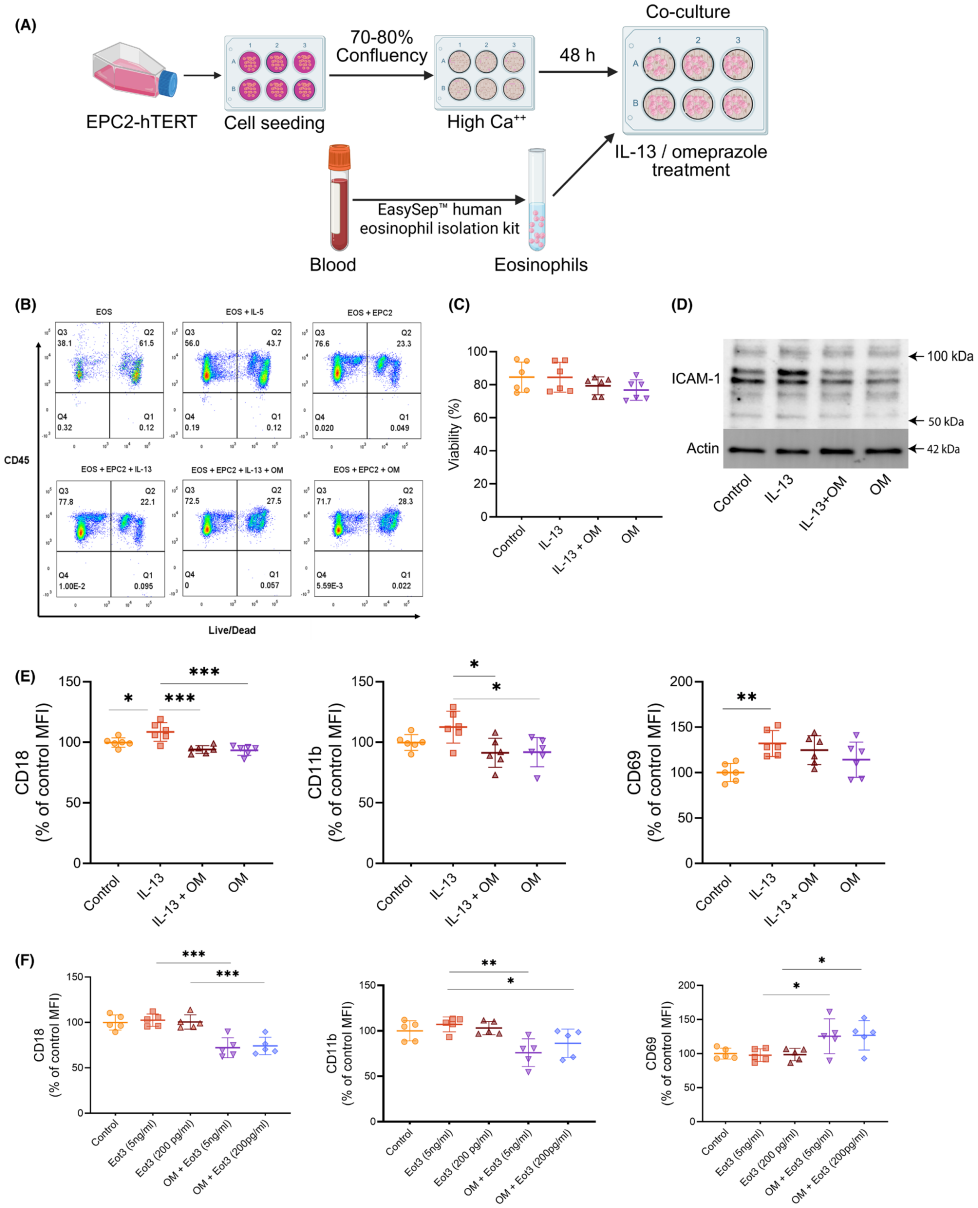
Omeprazole modulated eosinophil activation and adhesion marker expression in an eosinophil‐epithelial co‐culture model. (A) Schematic of EPC2‐hTERT esophageal epithelial and eosinophil co‐culture model. (B) Representative flow cytometry dot plots assessing eosinophil viability from cultures treated with or without IL‐5 or co‐cultured with EPC2‐hTERT cells. (C) Quantification of eosinophil viability in co‐culture under IL‐13 and/or omeprazole treatment (*n* = 6 biological replicates). (D) Representative western blot image showing ICAM‐1 expression in ALI cultures treated with IL‐13 and omeprazole. (E) Expression of eosinophil activation and adhesion proteins following omeprazole treatment in IL‐13‐stimulated epithelial‐eosinophil co‐cultures (mean ± SD, *n* = 6 biological replicates). (F) Effect of omeprazole on eosinophil activation markers in cultures supplemented with IL‐5 (5 ng/mL) and eotaxin‐3 (Eot3) (5 ng/mL and 200 pg/mL), presented as mean ± SD (*n* = 5 biological replicates). Statistical significance is indicated as follows: *p < .05, **<.01, ***<.001.

Given omeprazole's impact on epithelial chemokine secretion, we hypothesized that omeprazole treatment may affect eosinophil viability and activation by altering epithelial mediators. Eosinophils were isolated from whole blood and cultured with IL‐5 alone or co‐cultured with EPC2 cells for 24 h (Figure 5A), which was previously shown to prolong eosinophil survival and enhance viability.^24^ After culture, eosinophil viability was assessed via flow cytometry from the CD45+ population using a fixable viability dye (Figure 5B, Figure S5A). Eosinophil survival and activation were slightly higher in EPC2 co‐culture (median 96% vs. 67% viability, *p* = .029, Figure S5B,C) when compared to eosinophils cultured with IL‐5 alone. Adding IL‐13 or omeprazole to the co‐culture did not significantly alter viability compared to untreated EPC2 (Figure 4B,C).

We then examined eosinophil activation. Co‐cultured eosinophils treated with IL‐13 showed upregulated CD18 (*p* = .03), CD11b (*p* = .061), and CD69 (*p* = .008) compared to untreated control co‐cultures (Figure 5E). However, eosinophils treated with IL‐13 and omeprazole did not upregulate CD18, CD11b, or CD69 (Figure 5E). Additionally, ICAM‐1, the epithelial counter‐receptor for the CD11b/CD18 integrin complex, was upregulated in ALI with IL‐13 treatment but reduced when omeprazole was added (Figure 5D, Figure S6).

To determine whether omeprazole directly affected eosinophil activation and adhesion molecule expression, independent of chemokine levels in conditioned media, we examined eosinophil responses to defined concentrations of eotaxin‐3 (5 ng/mL or 200 pg/mL). Eosinophils were pretreated with omeprazole prior to eotaxin‐3 stimulation. Omeprazole‐pretreated eosinophils exhibited decreased CD18 and CD11b expression but increased CD69 expression following eotaxin‐3 treatment (Figure 5F), consistent with a direct effect of omeprazole on blocking eotaxin‐3‐induced CD11b and CD18 upregulation on eosinophils.

### 3.6 Omeprazole attenuated inflammatory cytokine secretion in co‐culture

**FIGURE 6:**
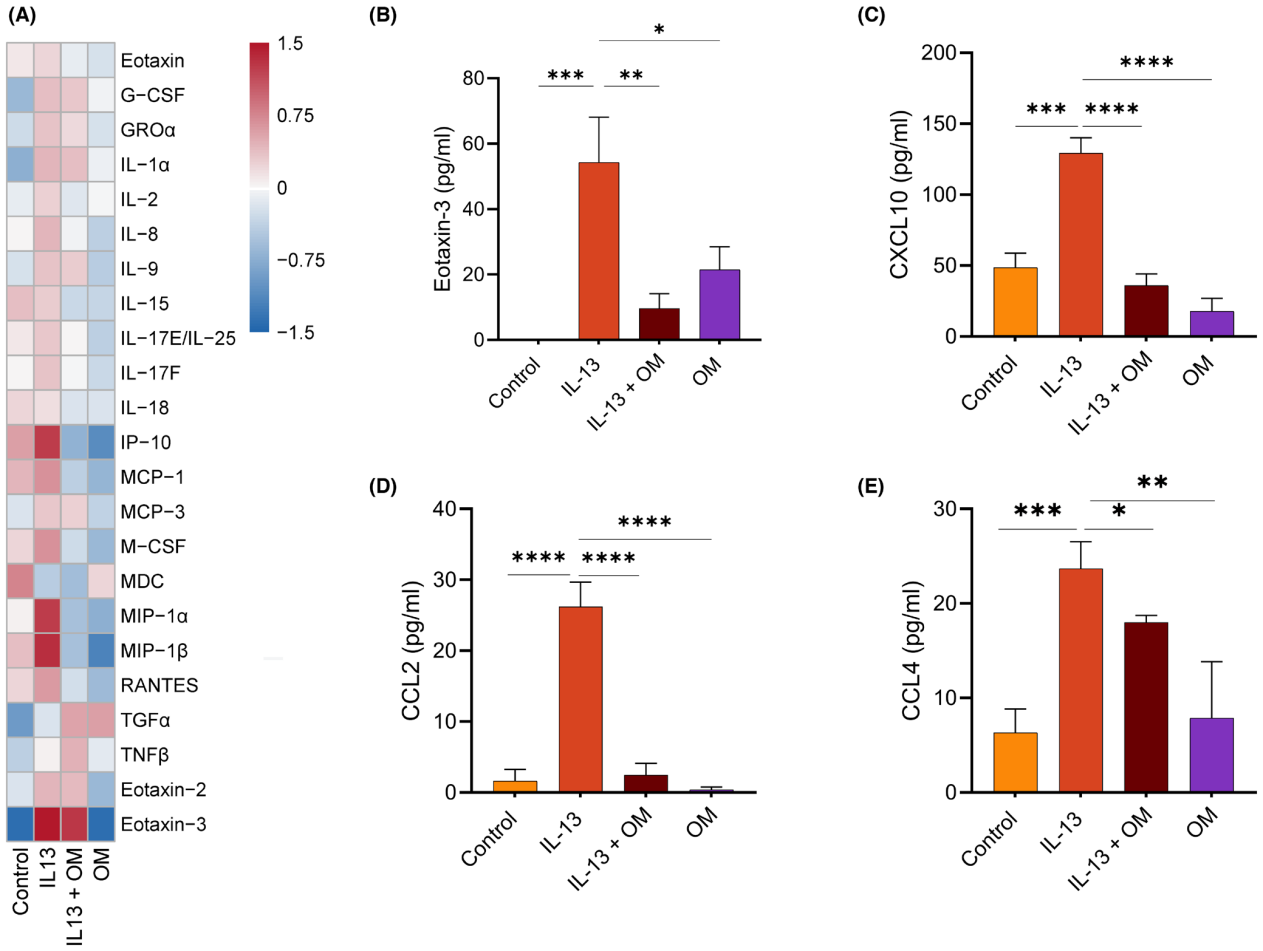
Omeprazole downregulated chemokine expression in IL‐13‐treated co‐culture of eosinophils and EPC2‐hTERT. (A) Heatmap depicting the average relative abundance of soluble mediators in co‐culture. Data were ln (*x* + 1) transformed and Pareto‐scaled to emphasize treatment specific patterns. (B) Eotaxin‐3 (*n* = 6), (C) CXCL10 (*n* = 5), (D) CCL2 (*n* = 5), (E) CCL4 (*n* = 4). Data are expressed as mean ± SD, and replicates are biological replicates. Statistical significance is indicated as follows: *p < .05, **p < .01, ***p < .001 and ****p < .0001.

Heatmap analysis of the multiplex assay highlighted major cytokines in the co‐culture (Figure 6A), showing that IL‐13 broadly upregulated inflammatory cytokine secretion, whereas omeprazole largely downregulated these responses. Based on these findings, we conducted ELISA assays to quantify soluble inflammatory markers. The overall pattern of eotaxin‐3 secretion was similar to that of the epithelial monoculture, as we observed a significant increase in eotaxin‐3 production with IL‐13 stimulation (54.27 ± 33.83 pg/mL) that was reduced with omeprazole treatment (9.633 ± 11.11 pg/mL) (Figure 6B). Furthermore, IL‐13 significantly elevated CXCL10 levels, which was attenuated by co‐stimulation with omeprazole or omeprazole alone (Figure 6C).

Although absent in the epithelial cell culture supernatant, monocyte chemoattractant protein‐1 (MCP‐1/CCL2) and macrophage inflammatory protein‐1β (MIP‐1β/CCL4) were observed in eosinophil‐epithelial co‐culture, and their expression was significantly upregulated by IL‐13. Additionally, treatment with IL‐13 and omeprazole alone led to a significant downregulation of these chemokines in the co‐culture system (Figure 6D,E), underscoring the anti‐inflammatory potential of omeprazole.

## 4 DISCUSSION

In recent years, PPIs have become a cornerstone treatment for EoE patients due to their accessibility, oral bioavailability, and favorable risk profile. Consequently, understanding the mechanisms driving their efficacy has become increasingly important. In the present study, we investigated the effects of omeprazole in the IL‐13 ALI model and identified distinct transcriptional patterns, with omeprazole modulating the expression of 39% of IL‐13‐responsive genes. This gene set was enriched in genes involved in epithelial differentiation and development, while other IL‐13–regulated genes remained largely unaffected.

Additionally, omeprazole disrupted key pathways that may affect eosinophil chemotaxis. Omeprazole treatment has previously been shown to disrupt esophageal epithelial transcription and secretion of CCL26. Here we show that other key epithelial signaling molecules, including CXCL10 and periostin, are decreased.^19^, ^20^, ^21^, ^22^, ^23^ Epithelial chemokines (eotaxin‐3, RANTES) and alarmins (IL‐33) upregulate eosinophil activation markers such as CD11b/CD18.^25^, ^26^, ^27^ Several studies have highlighted the role of the esophageal epithelium in providing regulatory signals to eosinophils within the mucosa,^24^, ^28^ including Dunn et al. who demonstrated that co‐culture of eosinophils and epithelial cells in the presence of IL‐13 enhanced eosinophil survival, upregulated eosinophil activation markers, and promoted a migratory eosinophil phenotype.^28^ Our data extend these findings by showing that in co‐cultures, omeprazole reduced IL‐13–induced eosinophil activation, as evidenced by decreased CD69 and integrin expression (CD11b/CD18). Prior studies in asthma and EoE have shown that the αMβ2 (CD11b/CD18) integrin is upregulated on eosinophils and plays a role in eosinophil migration and tissue persistence during allergic inflammation.^29^, ^30^, ^31^, ^32^ However, neither low (5 ng/mL) nor high (200 pg/mL) concentrations of eotaxin‐3 increased the expression of CD11b, CD18, or CD69. This suggests omeprazole may affect CD11b and CD18 expression through other mechanisms, since varying the eotaxin‐3 concentration within the ranges seen in our ELISA data from the enriched media did not alter CD11b and CD18 levels on eosinophils.

Integrins CD11b and CD18 play a key role in leukocyte migration to inflamed tissue by binding to ICAM‐1, which is upregulated on epithelial cells during inflammation^33^, ^34^ IL‐13 treatment increased epithelial ICAM‐1 expression, while co‐treatment with omeprazole reduced ICAM‐1 levels. Decreased ICAM‐1 expression may be another factor contributing to the reduced migration, adhesion, and activation of eosinophils observed in PPI‐responsive EoE. This is consistent with studies showing that pantoprazole decreased indomethacin‐induced ICAM‐1 in HUVEC cells.^35^ Together, our findings support a multifactorial mechanism by which PPIs disrupt epithelial–eosinophil interactions by decreasing the expression of epithelial adhesion molecules while also reducing the expression of leukocyte‐specific integrins on eosinophils.

Prior work by Zhang et al. suggested esophageal epithelium exhibited minimal changes in STAT6 phosphorylation with omeprazole treatment, but found a substantial decrease in STAT6 binding at the eotaxin‐3 promoter.^5^ Consistently, in C57BL/6 mouse‐derived esophageal 3D organoids, omeprazole reduced IL‐13‐induced STAT6 phosphorylation.^36^ Our data demonstrated a moderate reduction in STAT6 phosphorylation, as well as the ability of omeprazole to regulate key pathways, including differentiation and the expression of barrier proteins. Omeprazole partially restored IL‐13‐induced TEER disruption, FITC‐dextran permeability, histologic integrity, and reduced hyperplasia of TP63+ basal cells. Similar effects were observed in EoE patients by Rhijn et al., who found that PPI treatment partially improved the esophageal mucosal integrity as assessed by the presence of intracellular spaces, TEER, and transepithelial flux of small and large molecules.^37^ In contrast, Rochman et al. reported that ALI cultures treated with 100 μM PPI 2 days after IL‐13 (20 ng/mL) showed no change in TEER, whereas omeprazole given 1 h prior to IL‐13 (100 ng/mL) reduced Ki67^+^ cells, marker of cell proliferation.^4^ These differences highlight that in vitro assays, including the ALI model, are sensitive to conditions such as the timing and concentrations of stimulation. We administered acid‐activated omeprazole to ALI on the same day as IL‐13 treatment, and these different experimental conditions likely contribute to the varied TEER results observed between these experiments. Despite these differences, we saw similar effects on TP63, a proliferation marker, which aligns with previous findings of reduced Ki67^+^ cells in PPI‐treated ALI.

Although our study focused on specific treatment conditions, future research exploring additional time points, varying concentrations of IL‐13 and omeprazole, and other PPIs will be important for understanding the dose‐ and time‐dependent effects. These investigations could reveal whether similar pathways are modulated by different PPIs and help optimize PPI therapy for improved management of EoE. Moreover, because single‐nucleotide polymorphisms in STAT6 and CYP2C19 influence PPI response,^38^ genetic heterogeneity in patients cannot be captured by in vitro models alone.

We observed that omeprazole modulated genes implicated in epithelial barrier function. Downregulation of *DSG1* has been described in EoE patients in multiple transcriptional studies, and increased expression of the esophageal‐specific calpain 14 has been shown to reduce desmoglein‐1 expression, contributing to esophageal epithelial barrier dysfunction.^9^, ^10^ Omeprazole decreased expression of *CAPN14* and improved desmoglein‐1 expression, which may explain its ability to enhance TEER and preserve barrier integrity. This result is consistent with a previous transcriptome analysis of patients with PPI‐responsive EoE, where PPI therapy normalized the expression of cell–cell junction molecules, including *DSG1*, in patient biopsies.^3^ Furthermore, inhibition of pSTAT6 similarly downregulated *CAPN14* and upregulated desmoglein‐1, suggesting that omeprazole exerts these barrier‐protective effects via a STAT6‐dependent pathway. Desmoglein‐1 plays multiple roles in the epithelium by maintaining structural activity and inhibiting epithelial NF‐κB/ERBIN signaling.^39^

Our in vitro ALI and co‐culture models offer mechanistic insights into epithelial‐eosinophil interactions influenced by omeprazole but do not fully represent the complexity of the in vivo esophageal microenvironment, including other immune cells and the microbiome. Furthermore, the use of acid‐activated omeprazole in vitro does not fully replicate dynamic pharmacokinetic conditions expected in patients. Treatment timing, dosing, as well as patient genetic variability are additional factors that may influence the extent of PPI response seen in patients. Future studies using in vivo models and patient‐derived samples are needed to validate and extend these findings.

In summary, this study demonstrates that omeprazole modulates IL‐13‐induced transcriptional changes within the epithelium, improves epithelial barrier function via STAT6‐dependent pathways, and downregulates key chemokines and activation markers associated with eosinophilic inflammation. Together, these findings indicate that PPIs exert therapeutic effects in EoE not only through barrier restoration but also by reshaping epithelial–eosinophil crosstalk, highlighting this interaction as a central axis in mucosal immune regulation and a promising target for future therapies.

## AUTHOR CONTRIBUTIONS

**Ravi Gautam:** Conceptualization (equal); data curation (lead); formal analysis (equal); investigation (lead); methodology (lead); project administration (supporting); validation (lead); visualization (lead); writing – original draft (lead); writing – review and editing (equal). **Megha Lal:** Data curation (equal); formal analysis (equal); investigation (supporting); methodology (supporting); software (lead); visualization (supporting); writing – review and editing (supporting). **Margaret C. Carroll:** Data curation (supporting); methodology (supporting); writing – review and editing (supporting). **Zoe Mrozek:** Data curation (supporting); formal analysis (supporting); investigation (equal); methodology (supporting); visualization (supporting); writing – review and editing (supporting). **Tina Trachsel:** Formal analysis (supporting); investigation (supporting); writing – review and editing (supporting). **Jarad Beers:** Data curation (supporting); investigation (supporting); resources (supporting); writing – review and editing (supporting). **Melanie A. Ruffner:** Conceptualization (equal); formal analysis (supporting); funding acquisition (lead); investigation (equal); project administration (equal); resources (equal); supervision (lead); validation (supporting); visualization (supporting); writing – original draft (supporting); writing – review and editing (equal).

## FUNDING INFORMATION

Dr. Melanie Ruffner reports grant funding from the National Institutes of Health (NIH) K08AI148456, the American Academy of Allergy, Asthma & Immunology (AAAAI) Foundation, and the Margaret Q. Landenberger Research Foundation.

## CONFLICT OF INTEREST STATEMENT

The authors report no conflict of interest related to this work.

## Supporting information


Appendix S1.

## References

[1] Liacouras CA , Furuta GT , Hirano I , et al. Eosinophilic esophagitis: Updated consensus recommendations for children and adults. J Allergy Clin Immunol. 2011;128(1):3‐20. 21477849 10.1016/j.jaci.2011.02.040

[2] Dutta P , Shah‐Riar P , Bushra SS , et al. Recent trends in the Management of Eosinophilic Esophagitis: a systematic review. Cureus. 2023;15(8):e43221. 37692685 10.7759/cureus.43221 PMC10490439

[3] Wen T , Dellon ES , Moawad FJ , Furuta GT , Aceves SS , Rothenberg ME . Transcriptome analysis of proton pump inhibitor‐responsive esophageal eosinophilia reveals proton pump inhibitor‐reversible allergic inflammation. J Allergy Clin Immunol. 2015;135(1):187‐197. doi:10.1016/j.jaci.2014.08.043 25441638 PMC4289084

[4] Rochman M , Xie YM , Mack L , et al. Broad transcriptional response of the human esophageal epithelium to proton pump inhibitors. J Allergy Clin Immunol. 2021;147(5):1924‐1935. doi:10.1016/j.jaci.2020.09.039 33289661 PMC8062577

[5] Zhang X , Cheng E , Huo X , et al. Omeprazole blocks STAT6 binding to the eotaxin‐3 promoter in eosinophilic esophagitis cells. PLoS One. 2012;7(11):e50037. 23185525 10.1371/journal.pone.0050037 PMC3503709

[6] Cheng E , Zhang X , Huo X , et al. Omeprazole blocks eotaxin‐3 expression by oesophageal squamous cells from patients with eosinophilic oesophagitis and GORD. Gut. 2013;62(6):824‐832. doi:10.1136/gutjnl-2012-302250 22580413 PMC3552049

[7] Franciosi JP , Mougey EB , Dellon ES , et al. Proton pump inhibitor therapy for eosinophilic esophagitis: history, mechanisms, efficacy, and future directions. J Asthma Allergy. 2022;15:281‐302. doi:10.2147/JAA.S274524 35250281 PMC8892718

[8] Blanchard C , Mingler MK , Vicario M , et al. IL‐13 involvement in eosinophilic esophagitis: transcriptome analysis and reversibility with glucocorticoids. J Allergy Clin Immunol. 2007;120(6):1292‐1300. doi:10.1016/j.jaci.2007.10.024 18073124

[9] Davis BP , Stucke EM , Khorki ME , et al. Eosinophilic esophagitis–linked calpain 14 is an IL‐13–induced protease that mediates esophageal epithelial barrier impairment. JCI Insight. 2016;1(4):e86355. doi:10.1172/jci.insight.86355 27158675 PMC4855700

[10] Sherrill JD , Kc K , Wu D , et al. Desmoglein‐1 regulates esophageal epithelial barrier function and immune responses in eosinophilic esophagitis. Mucosal Immunol. 2014;7(3):718‐729. doi:10.1038/mi.2013.90 24220297 PMC3999291

[11] Molina‐Jiménez F , Ugalde‐Triviño L , Arias‐González L , et al. Proton pump inhibitor effect on esophageal protein signature of eosinophilic esophagitis, prediction, and evaluation of treatment response. Allergy. 2024;79(12):3448‐3463. doi:10.1111/all.16261 39092539 PMC11657045

[12] Ruffner MA , Song L , Maurer K , et al. Toll‐like receptor 2 stimulation augments esophageal barrier integrity. Allergy. 2019;74(12):2449‐2460. doi:10.1111/all.13968 31267532 PMC7083217

[13] Clough E , Barrett T , Wilhite SE , et al. NCBI GEO: archive for gene expression and epigenomics data sets: 23‐year update. Nucleic Acids Res. 2024;52(D1):D138‐D144. 37933855 10.1093/nar/gkad965 PMC10767856

[14] Kc K , Rothenberg ME , Sherrill JD . In vitro model for studying esophageal epithelial differentiation and allergic inflammatory responses identifies keratin involvement in eosinophilic esophagitis. PLoS One. 2015;10(6):e0127755. 26039063 10.1371/journal.pone.0127755 PMC4454568

[15] Clevenger MH , Karami AL , Carlson DA , et al. Suprabasal cells retain progenitor cell identity programs in eosinophilic esophagitis‐driven basal cell hyperplasia. JCI Insight. 2023;8(19):e171765. doi:10.1172/jci.insight.171765 37672481 PMC10619442

[16] Cortes JR , Rivas MD , Molina‐Infante J , et al. Omeprazole inhibits IL‐4 and IL‐13 signaling signal transducer and activator of transcription 6 activation and reduces lung inflammation in murine asthma. J Allergy Clin Immunol. 2009;124(3):607‐610.e1. doi:10.1016/j.jaci.2009.06.023 19665777

[17] Miller DE , Forney C , Rochman M , et al. Genetic, inflammatory, and epithelial cell differentiation factors control expression of human Calpain‐14. G3 Genes|Genomes|Genetics. 2019;9(3):729‐736. doi:10.1534/g3.118.200901 30626591 PMC6404614

[18] O'Shea KM , Aceves SS , Dellon ES , et al. Pathophysiology of eosinophilic esophagitis. Gastroenterology. 2018;154(2):333‐345. doi:10.1053/j.gastro.2017.06.065 28757265 PMC5787048

[19] Medoff BD , Sauty A , Tager AM , et al. IFN‐γ‐inducible protein 10 (CXCL10) contributes to airway hyperreactivity and airway inflammation in a mouse model of asthma. J Immunol. 2002;168(10):5278‐5286. doi:10.4049/jimmunol.168.10.5278 11994485

[20] Takaku Y , Nakagome K , Kobayashi T , Hagiwara K , Kanazawa M , Nagata M . IFN‐g‐inducible protein of 10 kDa upregulates the effector functions of eosinophils through b2 integrin and CXCR3. Respir Res. 2011;12:138. 22004287 10.1186/1465-9921-12-138 PMC3215664

[21] Blanchard C , Mingler MK , McBride M , et al. Periostin facilitates eosinophil tissue infiltration in allergic lung and esophageal responses. Mucosal Immunol. 2008;1(4):289‐296. doi:10.1038/mi.2008.15 19079190 PMC2683986

[22] Masuoka M , Shiraishi H , Ohta S , et al. Periostin promotes chronic allergic inflammation in response to Th2 cytokines. J Clin Invest. 2012;122(7):2590‐2600. doi:10.1172/JCI58978 22684102 PMC3386810

[23] Provost V , Larose MC , Langlois A , Rola‐Pleszczynski M , Flamand N , Laviolette M . CCL26/eotaxin‐3 is more effective to induce the migration of eosinophils of asthmatics than CCL11/eotaxin‐1 and CCL24/eotaxin‐2. J Leukoc Biol. 2013;94(2):213‐222. 23532518 10.1189/jlb.0212074

[24] Dunn JLM , Caldwell JM , Ballaban A , Ben‐Baruch Morgenstern N , Rochman M , Rothenberg ME . Bidirectional crosstalk between eosinophils and esophageal epithelial cells regulates inflammatory and remodeling processes. Mucosal Immunol. 2021;14(5):1133‐1143. doi:10.1038/s41385-021-00400-y 33972688 PMC8380647

[25] Chiba T , Kamada Y , Saito N , et al. RANTES and eotaxin enhance CD11b and CD18 expression on eosinophils from allergic patients with eosinophilia in the application of whole blood flow cytometry analysis. Int Arch Allergy Immunol. 2005;137(Suppl. 1):12‐16. 15947479 10.1159/000085426

[26] Suzukawa M , Koketsu R , Iikura M , et al. Interleukin‐33 enhances adhesion, CD11b expression and survival in human eosinophils. Lab Investig. 2008;88(11):1245‐1253. 18762778 10.1038/labinvest.2008.82

[27] Andreone S , Spadaro F , Buccione C , et al. IL‐33 promotes CD11b/CD18‐mediated adhesion of eosinophils to cancer cells and synapse‐polarized degranulation leading to tumor cell killing. Cancer. 2019;11(11):1664. 10.3390/cancers11111664 PMC6895824 31717819

[28] Dunn JL , Szep A , Gonzalez Galan E , et al. Eosinophil specialization is regulated by exposure to the esophageal epithelial microenvironment. J Leukoc Biol. 2024;116:qiae102. 10.1093/jleuko/qiae102 PMC11531809 38723185

[29] Barthel SR , Johansson MW , McNamee DM , Mosher DF . Roles of integrin activation in eosinophil function and the eosinophilic inflammation of asthma. J Leukoc Biol. 2008;83(1):1‐12. 17906117 10.1189/jlb.0607344 PMC2859217

[30] Johansson MW , Kelly EA , Busse WW , Jarjour NN , Mosher DF . Up‐regulation and activation of eosinophil integrins in blood and airway after segmental lung antigen challenge. J Immunol. 2008;180(11):7622‐7635. 18490765 10.4049/jimmunol.180.11.7622 PMC2585992

[31] Barthel SR , Jarjour NN , Mosher DF , Johansson MW . Dissection of the hyperadhesive phenotype of airway eosinophils in asthma. Am J Respir Cell Mol Biol. 2006;35(3):378‐386. 16601240 10.1165/rcmb.2006-0027OC PMC1550734

[32] Vimalathas P , Farris A , Letner D , et al. Integrin αM activation and upregulation on esophageal eosinophils and periostin‐mediated eosinophil survival in eosinophilic esophagitis. Immunol Cell Biol. 2018;96(4):426‐438. 29424023 10.1111/imcb.12018

[33] Diamond MS , Staunton DE , Marlin SD , Springer TA . Binding of the integrin mac‐1 (CD11b/CD18) to the third immunoglobulin‐like domain of ICAM‐1 (CD54) and its regulation by glycosylation. Cell. 1991;65(6):961‐971. 1675157 10.1016/0092-8674(91)90548-d

[34] Sumagin R , Robin AZ , Nusrat A , Parkos CA . Transmigrated neutrophils in the intestinal lumen engage ICAM‐1 to regulate the epithelial barrier and neutrophil recruitment. Mucosal Immunol. 2014;7(4):905‐915. 24345805 10.1038/mi.2013.106 PMC4062590

[35] Lee HJ , Han YM , Kim EH , Kim YJ , Hahm KB . A possible involvement of Nrf2‐mediated heme oxygenase‐1 up‐regulation in protective effect of the proton pump inhibitor pantoprazole against indomethacin‐induced gastric damage in rats. BMC Gastroenterol. 2012;12:1‐11. 23066659 10.1186/1471-230X-12-143 PMC3548718

[36] Morimoto M , Kawasaki K , McNamee N , et al. Mitochondrial dysfunction drives basal cell hyperplasia in eosinophilic oesophagitis. Gut. 2025;74(10):1571‐1588. doi:10.1136/gutjnl-2024-334561 40701793 PMC12438975

[37] van Rhijn BD , Weijenborg PW , Verheij J , et al. Proton pump inhibitors partially restore mucosal integrity in patients with proton pump inhibitor–responsive esophageal eosinophilia but not eosinophilic esophagitis. Clin Gastroenterol Hepatol. 2014;12(11):1815‐1823. 24657840 10.1016/j.cgh.2014.02.037

[38] Mougey EB , Williams A , Coyne AJK , et al. CYP2C19 and STAT6 Variants Influence the Outcome of Proton Pump Inhibitor Therapy in Pediatric Eosinophilic Esophagitis. J Pediatr Gastroenterol Nutr. 2019;69(5):581‐587. 31490856 10.1097/MPG.0000000000002480 PMC6855320

[39] Polivka L , Hadj‐Rabia S , Bal E , et al. Epithelial barrier dysfunction in desmoglein‐1 deficiency. J Allergy Clin Immunol. 2018;142(2):702‐706. 29705242 10.1016/j.jaci.2018.04.007 PMC6078820

