# Supplementary material for: Proton pump inhibitors modulate esophageal epithelial barrier function and crosstalk with eosinophils
Source: Pediatr Allergy Immunol. 2026 Feb 27;37(3):e70315. doi: 10.1111/pai.70315 (PMC12948656; doi:10.1111/pai.70315)
Supplement: Supplementary file 1 — Appendix S1. [file PAI-37-e70315-s001.pdf]

Online Repository

Proton pump inhibitors modulate esophageal epithelial barrier function and crosstalk with eosinophils

Ravi Gautam, PhD[1], Megha Lal, PhD[1], Margaret C. Carroll, BS[1], Zoe Mrozek, BS[1], Tina Trachsel, MD[1,3], Jarad Beers, MS[1], Melanie A. Ruffner, MD, PhD[1,2]

[1]Division of Allergy and Immunology, Children's Hospital of Philadelphia
[2]Department of Pediatrics, Perelman School of Medicine at University of Pennsylvania
[3] Division of Allergy, University Children's Hospital Zurich, Zurich, Switzerland.

Corresponding Author:
Melanie A. Ruffner, MD, PhD
Children's Hospital of Philadelphia
3615 Civic Center Blvd. ARC 1202
Philadelphia, PA 19104
ruffnerm@chop.edu

Supplemental Methods:

RNA-seq data analysis:

Quality assessment of sequencing reads was performed using FastQC 0.12.0 (1), and contaminant ribosomal RNA was filtered out using FastQ Screen 0.14.1 (2). The subsequent read alignment against GRCh38.p13 reference genome using STAR aligner 2.7.1a (3). The alignment files were then converted to BAM format, sorted, and indexed using samtools 1.12 (4). Post-alignment quality assessment was executed using Qualimap v2.2.1. Reads were then counted for exonic regions based on gene IDs utilizing the gencode.v29.annotation.gtf annotation file and featureCounts in subread-2.0.2 (5). Summary plots for the output files from the intermediate analysis were generated using MultiQC 1.10 (6). Subsequent downstream RNA-seq analysis was performed using R version 4.3.2.

Genes with counts less than 10 in at least three samples were removed to ensure robustness in the analysis. The gene expression data was normalized for sequencing depth and library composition using variance stabilizing transformation followed by differential gene expression analysis using DESeq2 1.44.0. Principal Component Analysis (PCA) was performed on all expressed genes as well as the 500 most variable genes to visualize sample clustering. The expression patterns were further visualized using a heatmap created with pheatmap 1.0.12. Gene identifiers in the published supplementary files were converted from Ensembl IDs to HGNC gene symbols using the Ensembl-HGNC Symbol Converter. Differential expression analysis involved estimating dispersions and applying Wald tests to determine statistical significance. Volcano plots were used to visualize differential expression across different conditions, and the Venn diagrams depicted meaningful biologically interpretable patterns, created using the VennDiagram package. Go-term enrichment analysis was conducted by clusterProfiler 4.12.0, which utilized Fisher's exact test to identify significant associations. Statistical significance was determined using the Benjamini-Hochberg (BH) method to correct for multiple testing.

Differential expression results from Rochman *et al.* were obtained directly from the published supplementary files and used to compare DEGs. The publicly available data from this study are reported as TPMs and were analyzed using an ANOVA-based framework, therefore we did not reanalyze expression values but extracted reported log2 fold changes and associated p-values. DEGs were compared between using a log fold change (logFC) threshold of ±1 and an adjusted p-value (padj) < 0.05 following identifier harmonization. LogFC values from the Rochman

supplementary data were compared with logFC values derived from the DESeq2 analysis of the in-house dataset. Directional concordance and relative effect sizes across studies was assessed.

REFERENCES

1. Andrews S. FastQC: a quality control tool for high throughput sequence data. Available online. *Retrieved May* 2010;**17**:2018.

2.Wingett SW, Andrews S. FastQ Screen: A tool for multi-genome mapping and quality control. *F1000Research* 2018;**7**.

3.Dobin A, Davis CA, Schlesinger F, Drenkow J, Zaleski C, Jha S et al. STAR: ultrafast universal RNA-seq aligner. *Bioinformatics* 2013;**29**:15–21.

4. Li H, Handsaker B, Wysoker A, Fennell T, Ruan J, Homer N et al. The sequence alignment/map format and SAMtools. *bioinformatics* 2009;**25**:2078–2079.

5. Liao Y, Smyth GK, Shi W. featureCounts: an efficient general purpose program for assigning sequence reads to genomic features. *Bioinformatics* 2014;**30**:923–930.

6.Ewels P, Magnusson M, Lundin S, Käller M. MultiQC: summarize analysis results for multiple tools and samples in a single report. *Bioinformatics* 2016;**32**:3047–3048.

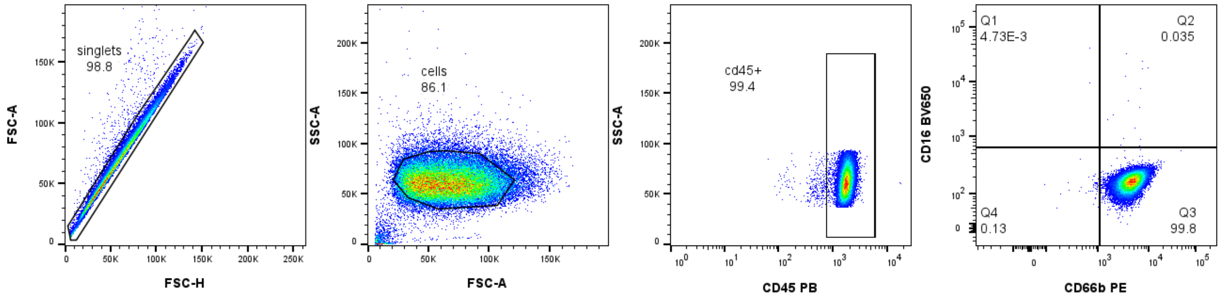

Supplemental Figure 1. The purity of eosinophils (CD16-CD66bCD45+) in the fraction obtained by using the EasySep human eosinophil isolation kit was analyzed by flow cytometry.

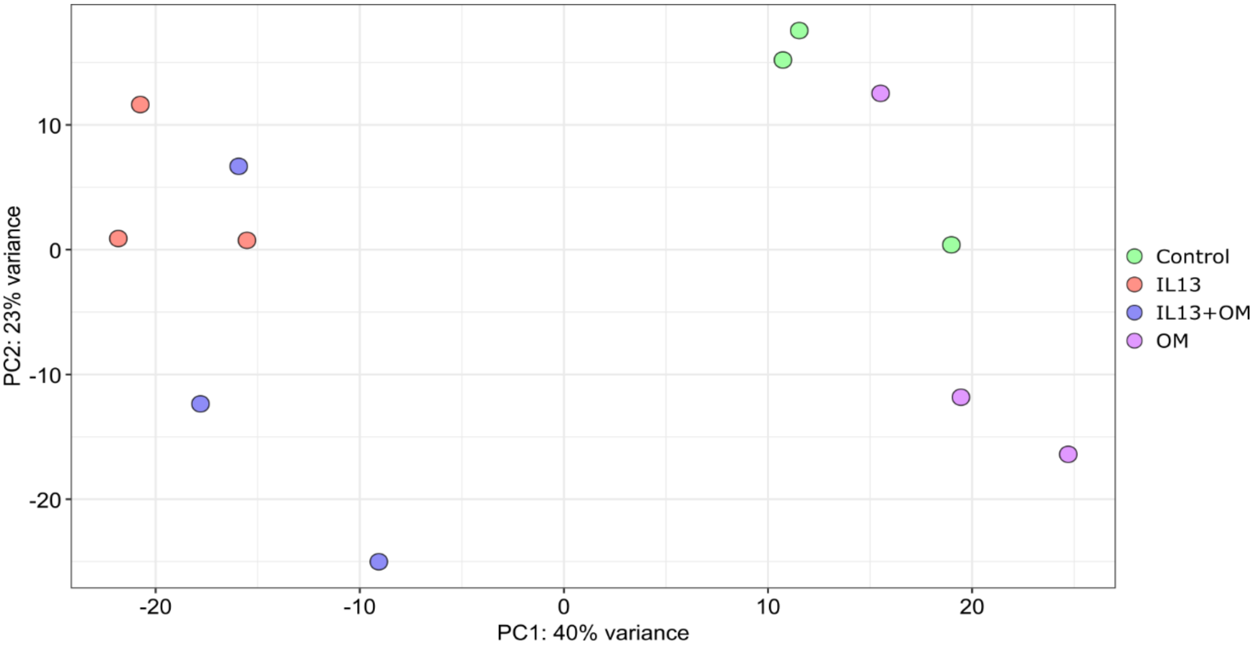

Supplemental Figure 2: PCA plot shows the distribution of gene expression profiles for the four experimental conditions: IL-13, Control, OM, and IL-13+OM.

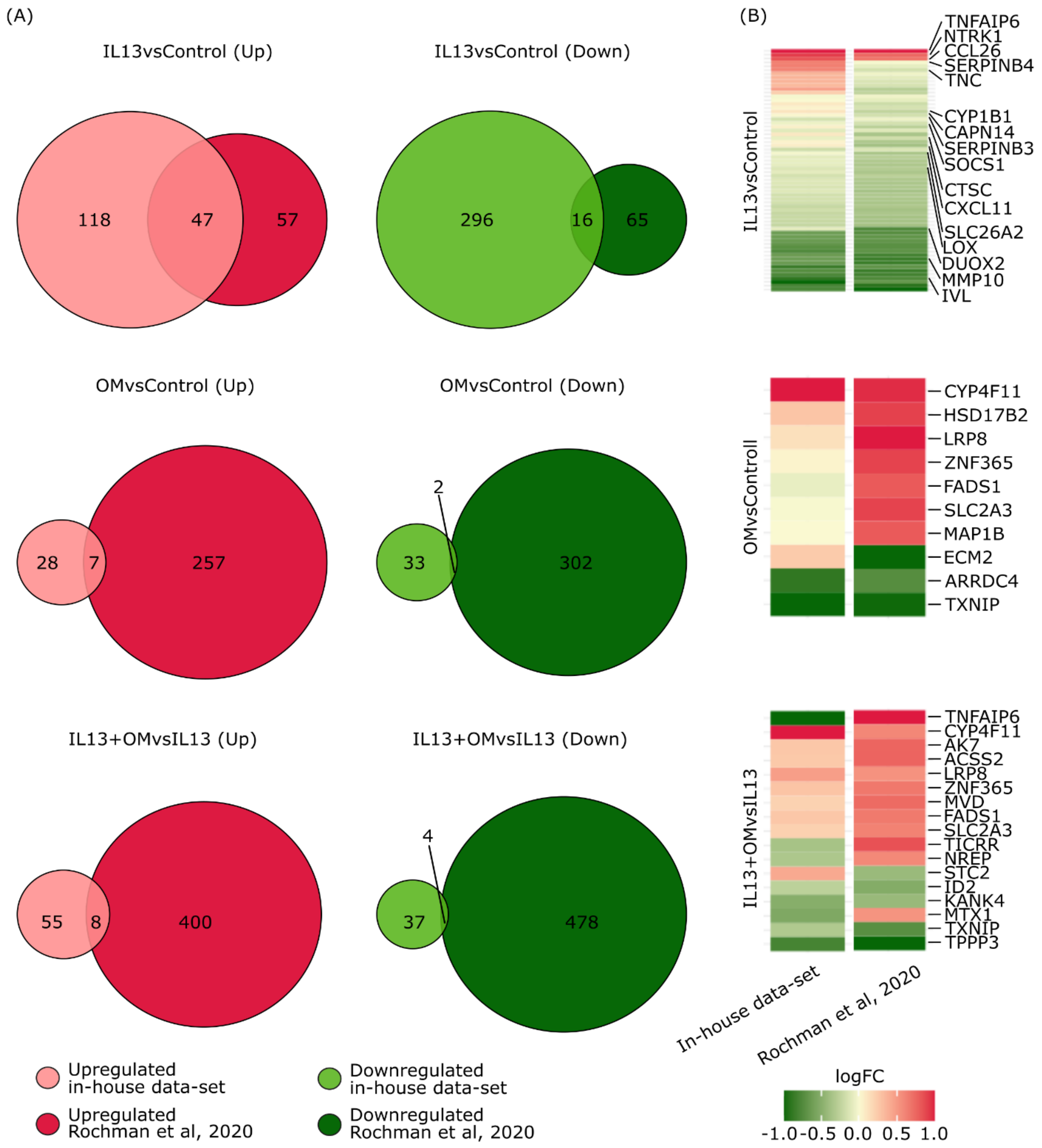

Supplemental Figure 3. Comparison of differentially expressed genes (DEGs) identified in this study and those reported by Rochman et al. (2021). Comparison of differentially expressed genes (DEGs) identified in this study and those reported by Rochman et al. (2021). Rochman et al. analyzed genes in submerged monolayer cells pre-treated with 100 µM omeprazole 1 hour prior to IL-13 (100 ng/ml) treatment for 24 hours. (A) Venn diagrams depict the numbers of shared and unique DEGs between the two datasets. (B) Heatmaps visualize expression patterns, highlighting similarities and differences derived from RNA-Seq analysis.

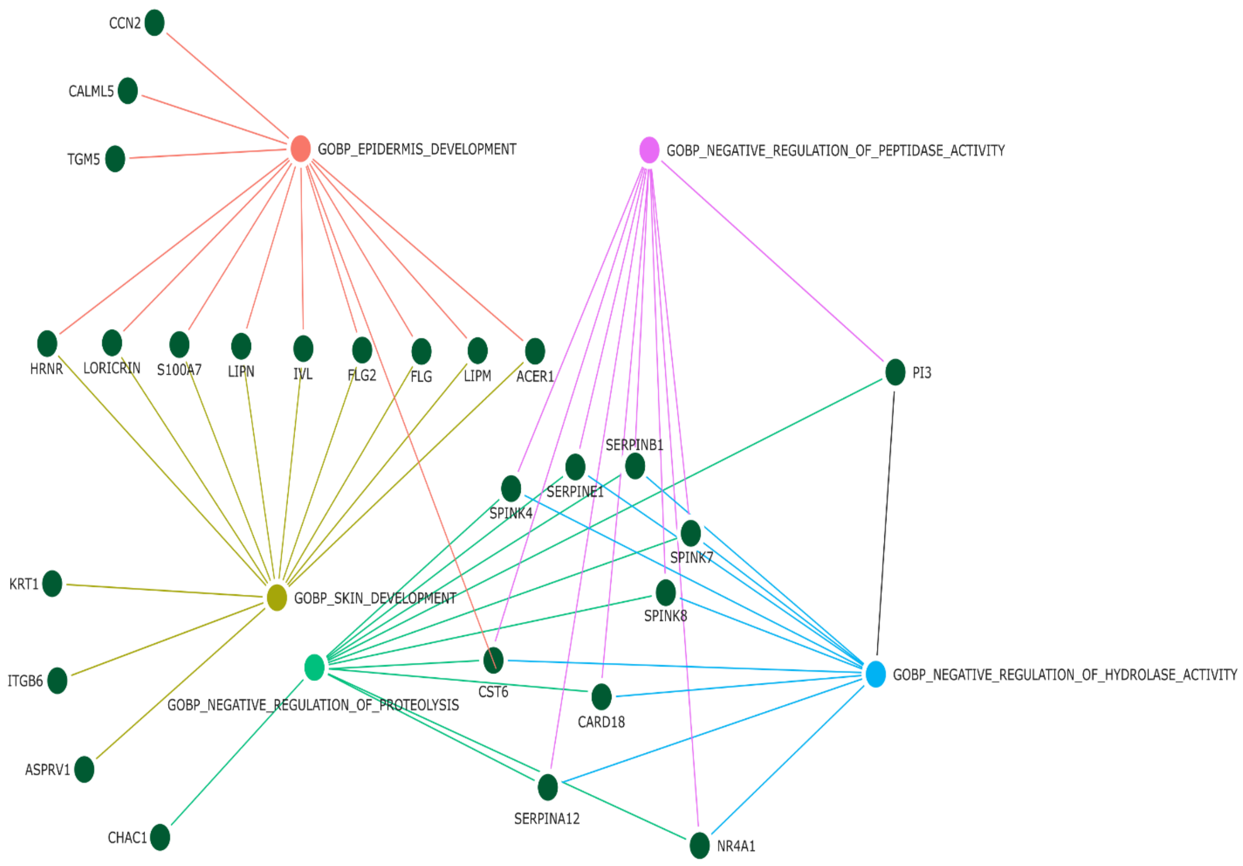

Supplemental Figure 4: GO term enrichment analysis of genes downregulated by IL-13 but not altered by omeprazole, highlighting associated pathways.

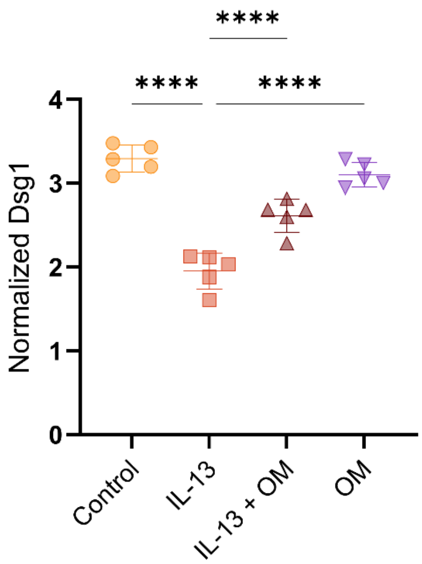

Supplemental Figure 5. Quantification of Dsg1 in ALI treated with IL-13 and omeprazole.

(A)

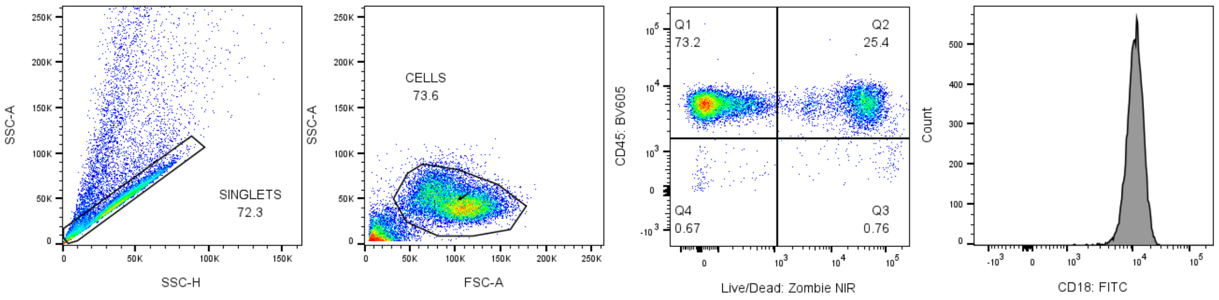

(B)

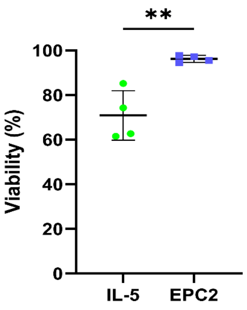

(C)

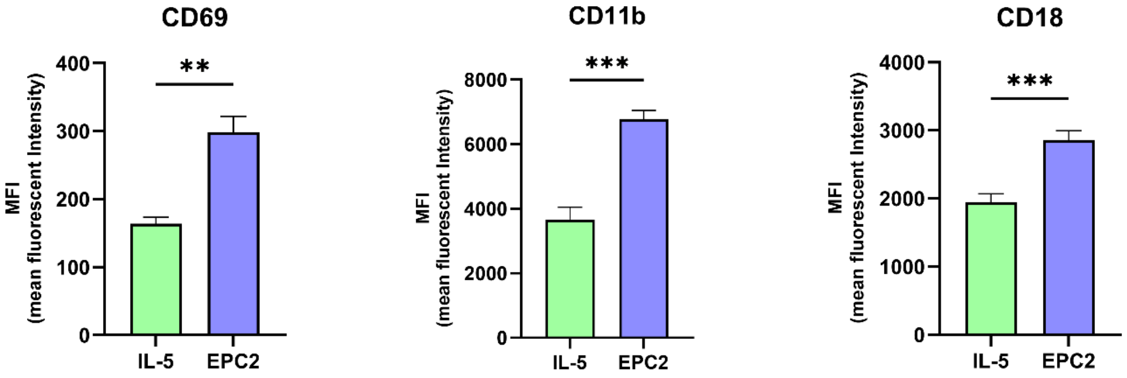

Supplemental Figure 5. Gating strategy and viability of eosinophils in co-culture. (A) Singlets were determined at first and the mean fluorescence intensity (MFI) was determined in live CD45+ cells. (B) percentage of total live eosinophils in culture with IL-5 (10 ng/ml) or EPC2 cells were determined following a similar gating strategy (C) Difference in activation markers of eosinophils in different culture conditions.

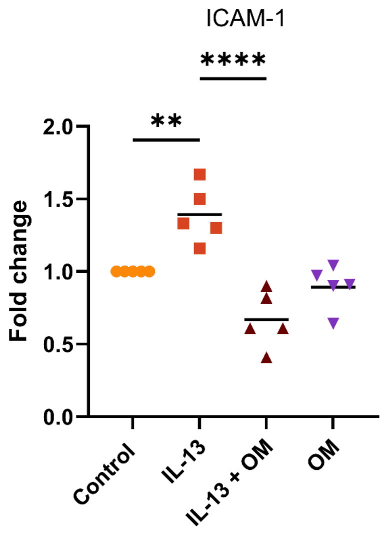

Supplemental Figure 6. Fold change of ICAM-1 expression relative to actin, calculated by the ratio of ICAM-1 to actin optical density, compared to control in ALI cultures.
